# Genome-wide analysis of porcine backfat and intramuscular fat fatty acid composition using high-density genotyping and expression data

**DOI:** 10.1186/1471-2164-14-845

**Published:** 2013-12-02

**Authors:** María Muñoz, M Carmen Rodríguez, Estefânia Alves, Josep María Folch, Noelia Ibañez-Escriche, Luis Silió, Ana Isabel Fernández

**Affiliations:** INIA, Mejora Genética Animal, 28040 Madrid, Spain; Centro I + D en Cerdo Ibérico-INIA, 06300 Zafra (Badajoz), Spain; Centre de Recerca en Agrigenòmica (CRAG), Consorci CSIC-IRTA-UAB-UB, Campus UAB, Bellaterra, 08193 Spain; Departament de Ciència Animal i dels Aliments, Facultat de Veterinària, Universitat Autònoma de Barcelona, 08193 Bellaterra, Spain; IRTA, Genètica y Mejora Animal, 25198 Lleida, Spain

**Keywords:** Fatty acid composition, Pleiotropic effects, QTL scan, eQTL

## Abstract

**Background:**

Porcine fatty acid composition is a key factor for quality and nutritive value of pork. Several QTLs for fatty acid composition have been reported in diverse fat tissues. The results obtained so far seem to point out different genetic control of fatty acid composition conditional on the fat deposits. Those studies have been conducted using simple approaches and most of them focused on one single tissue. The first objective of the present study was to identify tissue-specific and tissue-consistent QTLs for fatty acid composition in backfat and intramuscular fat, combining linkage mapping and GWAS approaches and conducted under single and multitrait models. A second aim was to identify powerful candidate genes for these tissue-consistent QTLs, using microarray gene expression data and following a targeted genetical genomics approach.

**Results:**

The single model analyses, linkage and GWAS, revealed over 30 and 20 chromosomal regions, 24 of them identified here for the first time, specifically associated to the content of diverse fatty acids in BF and IMF, respectively. The analyses with multitrait models allowed identifying for the first time with a formal statistical approach seven different regions with pleiotropic effects on particular fatty acids in both fat deposits. The most relevant were found on SSC8 for C16:0 and C16:1(n-7) fatty acids, detected by both linkage and GWAS approaches. Other detected pleiotropic regions included one on SSC1 for C16:0, two on SSC4 for C16:0 and C18:2, one on SSC11 for C20:3 and the last one on SSC17 for C16:0. Finally, a targeted eQTL scan focused on regions showing tissue-consistent effects was conducted with *Longissimus* and fat gene expression data. Some powerful candidate genes and regions were identified such as the *PBX1, RGS4*, *TRIB3* and a transcription regulatory element close to *ELOVL6* gene to be further studied.

**Conclusions:**

Complementary genome scans have confirmed several chromosome regions previously associated to fatty acid composition in backfat and intramuscular fat, but even more, to identify new ones. Although most of the detected regions were tissue-specific, supporting the hypothesis that the major part of genes affecting fatty acid composition differs among tissues, seven chromosomal regions showed tissue-consistent effects. Additional gene expression analyses have revealed powerful target regions to carry the mutation responsible for the pleiotropic effects.

**Electronic supplementary material:**

The online version of this article (doi:10.1186/1471-2164-14-845) contains supplementary material, which is available to authorized users.

## Background

Dietary fatty acids (FA) are highly relevant for human health, since some saturated FA (SFA) of medium chain length such as lauric (C12:0) and myristic (C14:0) increase the plasmatic cholesterol level and the risk of cardiovascular diseases [1]. A protective effect reducing cholesterol in blood is attributed to polyunsaturated FA, but from a nutritional point of view the balance between polyunsaturated and monounsaturated FA (PUFA/MUFA) and the n-6/n-3 PUFA ratio are more appreciated than the content of particular fatty acids [2]. It is also well known that fat and long-chain FA, whether in adipose tissue or muscle, affect sensorial and technological properties of meat and meat products [3]. The FA content of pig meat products influences their tenderness, juiciness and flavor [4]. For instance, the pleasant flavor associated to Iberian pig dry-cured hams with high levels of oleic acid and MUFA is explained by the presence in these products of high levels of oleic acid-derived volatiles [5].

The FA composition of porcine fat and muscle tissues exhibits moderate to high heritability values, and the results of studies performed on data from several tissues point out the different genetic control of FA composition in diverse fat and muscle tissues [6–8]. Classical studies of QTL detection, based on microsatellites genotyping data from experimental crosses, have reported several QTL affecting FA composition in different porcine tissues [9–14], and a few significant QTLs in SSC4 [10], SSC7 [14] and SSC14 [13] affecting the content in diverse fat locations of a particular FA. However, only single-trait single-QTL models were used for the corresponding statistical analyses. More complex multitrait models are required for a rigorous checking of pleiotropic QTL effects and for increasing the precision of its estimated location in the genome [15].

The availability of the Illumina PorcineSNP60 BeadChip [16] has improved the genetic analyses of complex traits through the use of high-density genotyping markers. Some studies with high-density chips using the Genome Wide Association approach (GWAS) have been carried out for detecting QTLs affecting FA content [11, 17]. Other studies based on classical models accounting for linkage allowed fine-mapping of significant QTLs not observed by GWAS [18]. Moreover, the integration of the results of QTL fine-mapping with microarray expression data offers a promising tool for understanding the genetic mechanisms influencing complex traits. The expression level of each probe may be treated as a quantitative trait and the marker genotypes used to map loci with regulatory effect on the gene expression level (eQTL). So far, only three global analyses have been published for pigs. In these studies, the combination of genome scans for phenotypic QTL (pQTL) and eQTL has provided key positional candidate genes for important complex pig phenotypes [19–21]. Some variants of this general approach, such as targeted genetical genomics, have been proposed to reduce its high cost [22].

In the present study, an analysis of the genetic basis of the FA composition of backfat (BF) and intramuscular fat (IMF) was performed in an experimental backcross between divergent pig lines with the following objectives; 1) to identify significant QTLs for these traits from two complementary genome scans based on linkage mapping and GWAS, 2) to determine pleiotropic QTLs affecting the content of particular FA in both tissues, 3) to propose candidate genes explaining the putative pleiotropic regions combining targeted eQTL and eGWAS approaches.

## Results

**Figure 1:**
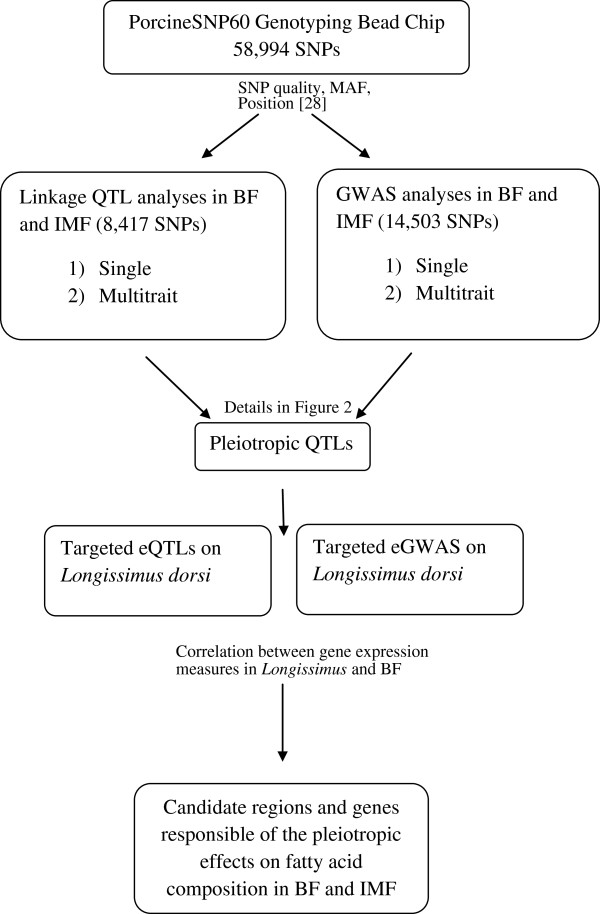
**Work flow representation followed in the current study.**

In the current study, QTL scans by linkage and GWAS have been conducted in order to identify chromosomal regions with significant tissue-specific and tissue-consistent effects on the FA composition of two fat deposits, IMF and BF. An additional targeted eQTL analysis focused on regions showing tissue-consistent effects was conducted to identify powerful candidate genes underlying these pleiotropic effects (Figure 1).

### *Linkage QTL scan*

Different linkage QTL scans, using single and multitrait models fitting diverse QTL effects, were carried out in order to identify tissue-specific or tissue-consistent QTL for FA composition of backfat and intramuscular fat. The performed QTL scans were conditional on the genotyping data of 8,417 SNPs evenly distributed along each chromosome. The single QTL scan for FA composition in BF revealed at least 11 significant QTLs, taking into account the test multiplicity (*q-*value < 0.05) with effects on the percentages of C14:0, C16:0, C16:1(n-7), C16:1(n-9), C17:0, C18:2, C18:3(n-6) and C20:3 fatty acids in nine porcine autosomes (SSC4, SSC7, SSC8, SSC11, SSC12, SSC14, SSC15, SSC16 and SSC17) (Table 1). Furthermore, at least 14 significant QTLs (*q-*value < 0.05) with effects on the percentages in IMF of C14:0, C16:0, C16:1(n-7), C18:0, C18:2, and C20:3 were identified in nine porcine autosomes (SSC3, SSC4, SSC7, SSC8, SSC10, SSC11, SSC14, SSC16 and SSC17) (Table 2).

**Table 1:** **Significant QTLs affecting BF fatty acid composition (FDR = 0.05)**

SSC	Trait	Position (CI)	LR	***P***-value	***a*** (SE)	Ref^§^
4	C16:0_BF_	61 (61–62)	10.19	1.8 × 10^-3^	0.52 (0.16)	[11, 13, 23]
	C16:1(n-9)_BF_	61 (60–62)	26.76	2.9 × 10^-3^	−0.05 (0.01)	[11, 23]
	C18:2_BF_	61 (62–69)	25.70	5.5 × 10^-4^	−1.16 (0.22)	[9, 10, 23, 24]
	C18:3(n-6)_BF_	61 (61–62)	16.34	2.8 × 10^-3^	−0.06 (0.01)	[14]
	C20:3_BF_	75 (75–81)	18.50	1.6 × 10^-3^	−0.05 (0.01)	[14]
7	C17:0_BF_	8 (7–10)	12.47	4.1 × 10^-5^	−0.05 (0.01)	New
8	C14:0_BF_	91 (86–92)	27.46	3.8 × 10^-4^	0.07 (0.01)	New
	C16:0_BF_	89 (85–90)	41.77	1.8 × 10^-3^	0.95 (0.14)	[9, 12]
	C16:1(n-7)_BF_	90 (86–90)	25.26	2.9 × 10^-3^	0.18 (0.03)	[9, 11, 25]
	C20:3_BF_	86 (85–90)	15.01	0.6 × 10^-3^	−0.04 (0.01)	New
11	C17:0_BF_	10 (9–16)	15.53	2.8 × 10^-3^	0.05 (0.01)	New
12	C14:0_BF_	39 (37–41)	12.30	1.6 × 10^-3^	0.05 (0.01)	[13, 26, 27]
	C16:0_BF_	66 (60–66)	9.20	4.1 × 10^-5^	0.50 (0.16)	New
	C17:0_BF_	1 (1–3)	12.45	3.8 × 10^-4^	0.05 (0.01)	New
14	C16:0_BF_	13 (12–15)	8.55	1.8 × 10^-3^	0.47 (0.16)	[12]
15	C16:1(n-7)_BF_	6 (5–10)	10.78	2.9 × 10^-3^	−0.12 (0.04)	[11]
16	C16:1(n-7)_BF_	15 (10–17)	8.74	5.5 × 10^-4^	−0.11 (0.03)	New
17	C16:0_BF_	58 (58–62)	7.68	2.8 × 10^-3^	0.48 (0.17)	New
	C20:3_BF_	60 (58–62)	10.17	1.4 × 10^-3^	−0.03 (0.01)	New

Position: cM, CI: confidence intervals; LR: Likelihood ratio test values; *a* (SE): additive effect (standard error); ^§^Reference number of the previous studies identifying a QTL for the same trait in any fat tissue.

**Table 2:** **Significant QTLs affecting IMF fatty acid composition (FDR = 0.05)**

SSC	Trait	Position (CI)	LR	***P***-value	***a*** (SE)	Ref^§^
3	C18:0_IMF_	5 (4–8)	16.19	5.7 × 10^-5^	0.61 (0.15)	New
	C18:2_IMF_	12 (11–12)	11.29	7.8 × 10^-4^	−1.00 (0.35)	New
4	C16:1(n-7)_IMF_	104 (104–110)	13.58	2.3 × 10^-4^	0.20 (0.05)	[11]
	C18:2_IMF_	40 (31–44)	21.27	4.0 × 10^-6^	−1.49 (0.33)	[9, 10, 23, 24]
	C20:3_IMF_	40 (34–47)	13.65	2.2 × 10^-4^	−0.08 (0.02)	[14]
7	C18:2_IMF_	121 (103–128)	11.64	6.5 × 10^-4^	0.99 (0.35)	[11]
8	C16:0_IMF_	96 (86–97)	18.09	2.1 × 10^-5^	0.69 (0.16)	[9, 11, 12]
	C16:1(n-7)_IMF_	90 (84–90)	20.20	7.0 × 10^-6^	0.23 (0.05)	[9, 11, 25]
	C18:2_IMF_	98 (98–99)	8.98	2.7 × 10^-3^	−0.76 (0.35)	[25]
10	C18:2_IMF_	81 (79–82)	9.72	1.8 × 10^-3^	−0.83 (0.36)	[12, 13]
11	C18:2_IMF_	1 (1–8)	8.90	2.9 × 10^-3^	0.79 (0.36)	[11]
	C20:3_IMF_	7 (2–8)	11.95	5.5 × 10^-3^	0.07 (0.02)	New
14	C18:2_IMF_	4 (3–4)	8.93	2.8 × 10^-3^	−0.75 (0.35)	[11–13]
16	C18:2_IMF_	70 (69–75)	9.91	1.6 × 10^-3^	0.84 (0.34)	[11]
17	C14:0_IMF_	58 (58–64)	16.83	4.1 × 10^-5^	0.09 (0.02)	[11]
	C16:0_IMF_	58 (57–62)	12.63	3.8 × 10^-4^	0.61 (0.17)	New

Position: cM, CI: confidence intervals; LR: Likelihood ratio test values; *a* (SE): additive effect (standard error); ^§^Reference number of the previous studies identifying a QTL for the same trait in any fat tissue.

**Figure 2:**
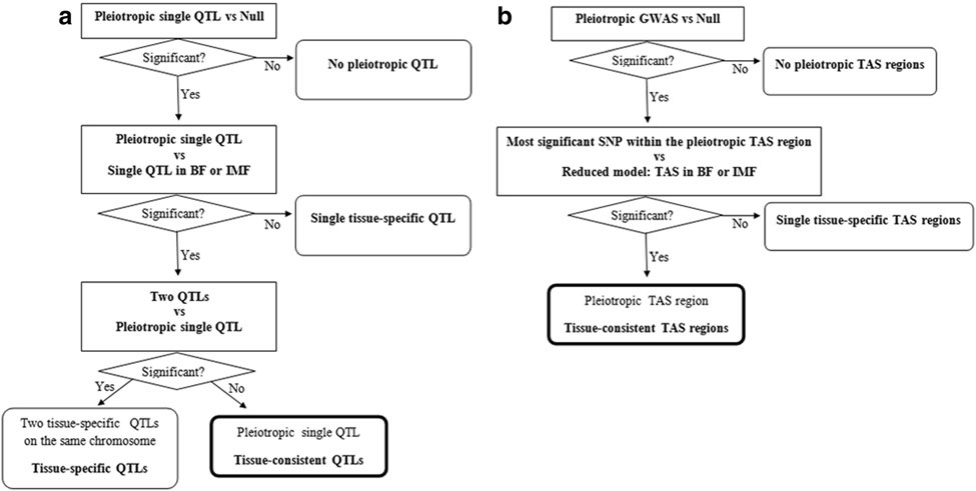
**Decision tree used for identifying pleiotropic QTL and TAS regions. a)** Decision tree used to identify pleiotropic linkage QTL for a particular fatty acid in both IMF and BF fat deposits; **b)** Decision tree used for identifying pleiotropic TAS regions for a particular fatty acid in both IMF and BF fat deposits.

Pleiotropic QTLs with consistent effects on both tissues were identified following the decision tree of the statistical contrasts shown in Figure 2a. Those putative pleiotropic QTLs (*q-*value < 0.05) identified in a QTL genome scan using a model fitting pleiotropic effects on a particular FA in both fat depots, were tested in a second step against reduced bivariate models fitting one single QTL effect on BF or IMF. Subsequently, the pleiotropic QTL that remained significant were tested against a model considering two different QTLs on the same chromosome, one for each tissue. The results revealed two highly significant pleiotropic QTL regions in SSC8 at 87 cM for C16:0_BF_ and C16:0_IMF_ and at 90 cM for C16:1(n-7)_BF_ and C16:1(n-7)_IMF_ (Table 3). Other pleiotropic QTL regions were also identified in the SSC11 at 7 cM for C20:3_BF_ and C20:3_IMF_ and at 58 cM in SSC17 for C16:0_BF_ and C16:0_IMF_ (Table 3). However, the results of LR tests indicated that models fitting two QTLs by chromosome were more likely than the putative pleiotropic QTL for C16:0_,_ C18:2 and C20:3 mapping in SSC4, and for C16:0 in SSC12 (Table 3). Moreover, as several QTL mapped at closed positions on SSC8 (Tables 1, 2 and 3), multitrait models including different combinations of FA were used to refine the QTL positions. The results supported the presence on SSC8 of the pleiotropic QTL affecting C14:0_BF_ , C16:0_BF_ , C16:1(n-7)_BF_ , C16:0_IMF_ and C16:1(n-7)_IMF_ at 90 cM (nominal *P-*value = 0.2 × 10^-10^) and two QTLs with effect on C20:3_BF_ and C18:2_IMF_ located at 86 and 96 cM respectively.

**Table 3:** **Results of pleiotropic QTL scan and testing of models fitting one or two QTLs per chromosome**

SSC	Trait	Position (CI)	LR	***P-***value	***a*** (SE)
Most likely model: one pleiotropic QTL					
8	C16:0_BF_	87 (87–88)	52.34	4.3 × 10^-12^	0.94 (0.13)
	C16:0_IMF_				0.65 (0.15)
	C16:1(n-7)_BF_	90 (86–91)	32.99	6.8 × 10^-8^	0.18 (0.03)
	C16:1(n-7)_IMF_				0.23 (0.05)
11	C20:3_BF_	7 (7–8)	15.91	3.5 × 10^-4^	0.02 (0.01)
	C20:3_IMF_				0.07 (0.02)
17	C16:0_BF_	58 (58–59)	15.54	4.2 × 10^-4^	0.45 (0.16)
	C16:0_IMF_				0.57 (0.16)
Most likely model: two QTLs on the same chromosome					
4	C16:0_BF_	61 (61–64)	19.70	1.9 × 10^-4^	0.49 (0.13)
	C16:0_IMF_	104 (101–104)			0.43 (0.13)
	C18:2_BF_	61 (61–62)	37.99	5.6 × 10^-9^	−1.18 (0.21)
	C18:2_IMF_	35 (34–45)			−1.46 (0.33)
	C20:3_BF_	75 (75–77)	35.64	8.9 × 10^-8^	−0.05 (0.01)
	C20:3_IMF_	43 (42–46)			−0.08 (0.02)
12	C16:0_BF_	68 (66–70)	24.43	2.0 × 10^-5^	0.63 (0.13)
	C16:0_IMF_	34 (33–38)			0.65 (0.14)

All the QTL reached the FDR < 0.05; Position: cM, CI: Confidence intervals; LR: Likelihood ratio test values; *a* (SE): additive effect (standard error).

### *GWAS scan*

Similarly to the linkage QTL analyses, single and bivariate GWAS were performed in order to identify regions with tissue-specific or tissue-consistent effects on FA composition. The GWAS was conducted using a subset of 14,503 SNPs from the PorcineSNP60 Genotyping Bead Chip (Illumina) previously selected for building a high-density linkage map [28]. The single association analysis revealed a total of 515 and 495 SNPs statistically associated to the content of some FA in BF and IMF, respectively. Those chromosomal regions containing more than two significant SNPs (*q*-value < 0.05) not fully linked and with a distance between contiguous SNPs lower than 1.5 cM, were set up as significant trait-associated SNPs (TAS) regions. A total of 44 TAS regions, containing 395 TAS, distributed across SSC1, SSC4, SSC6, SSC8, SSC11, SSC12, SSC13, SSC14, SSC15, SSC16 and SSC18, showed association with FA composition of BF (Table 4). In addition, 35 TAS regions, containing 381 TAS, distributed across SSC1, SSC4, SSC5, SSC8, SSC11, SSC13, SSC14, SSC15 and SSC17, showed association with FA composition of IMF (Table 5).

**Table 4:** **Chromosomal Regions with Trait Associated SNPs (TAS) affecting BF fatty acid (FDR = 0.05)**

SSC	Trait	Chromosomal region (Mb)	N° of TAS	***P***-value	***a*** (SE)	Ref^§^
1	C16:0_BF_	38.33-40.62	10	2.3 × 10^-4^	−0.71 (0.19)	[11]
		82.70-84.02	17	3.2 × 10^-4^	−0.55 (0.15)	[11]
		135.93-136.24	2	7.2 × 10^-4^	−0.51 (0.15)	[11]
	C20:3_BF_	48.24-49.05	4	5.8 × 10^-5^	−0.03 (0.01)	New
4	C16:0_BF_	44.98-45.19	5	3.1 × 10^-5^	−0.73 (0.17)	[11, 13, 23]
		97.76-98.84	4	7.7 × 10^-4^	−0.49 (0.14)	[11, 13, 23]
		111.83-112.18	2	3.0 × 10^-1^	−0.50 (0.14)	[11]
	C16:1(n-9)_BF_	41.61-44.10	9	1.1 × 10^-7^	−0.04 (0.01)	[11]
		46.82-46.86	2	6.6 × 10^-5^	−0.02 (0.01)	[11]
		59.06-62.21	10	1.6 × 10^-5^	0.05 (0.01)	[11, 23]
		68.05-94.49	41	1.2 × 10^-6^	−0.04 (0.01)	[11, 23]
		95.37-98.25	7	1.3 × 10^-6^	−0.04 (0.01)	[11]
		102.45-103.74	5	2.8 × 10^-5^	−0.03 (0.01)	[11]
		106.41-110.55	14	2.3 × 10^-5^	0.03 (0.01)	[11]
		124.78-124.83	2	1.1 × 10^-5^	−0.03 (0.01)	[11]
	C18:2_BF_	89.10-92.44	8	3.1 × 10^-6^	−0.90 (0.18)	[9, 23, 24]
	C20:3_BF_	94.49-100.44	10	1.4 × 10^-6^	−0.04 (0.01)	[14]
		102.45-102.51	3	5.9 × 10^-5^	−0.03 (0.01)	[14]
6	C14:0_BF_	8.01-8.06	2	1.1 × 10^-4^	−0.05 (0.01)	New
8	C14:0_BF_	99.33-99.49	3	8.1 × 10^-7^	0.05 (0.01)	New
		110.90-125.08	28	7.8 × 10^-11^	−0.07 (0.01)	New
	C16:0_BF_	27.62-28.84	5	1.8 × 10^-4^	−0.48 (0.12)	[13]
		40.85-43.06	5	6.0 × 10^-7^	−0.75 (0.14)	[9, 12]
		63.12-65.55	6	1.2 × 10^-6^	0.57 (0.11)	[9, 12, 25]
		75.13-78.81	8	1.8 × 10^-8^	−0.72 (0.12)	[9, 12, 25]
		83.84-126.88	102	1.0 × 10^-17^	−1.00 (0.10)	[9, 11, 12]
		130.63-131.42	3	2.6 × 10^-9^	0.75 (0.12)	[11]
	C16:1(n-7)_BF_	99.33-99.49	3	1.1 × 10^-6^	0.13 (0.03)	[9, 11, 25]
		110.90-125.08	27	2.6 × 10^-9^	−0.16 (0.02)	[9, 11, 25]
	C20:3_BF_	91.57-92.43	4	5.7 × 10^-5^	0.03 (0.01)	New
		99.33-99.49	4	4.9 × 10^-6^	−0.03 (0.01)	New
		117.67-120.10	6	5.6 × 10^-6^	0.03 (0.01)	New
		122.07-124.10	4	2.0 × 10^-5^	0.06 (0.01)	New
11	C16:0_BF_	23.15-23.20	2	9.3 × 10^-4^	0.43 (0.11)	New
		81.98-82.21	2	7.8 × 10^-4^	−0.51 (0.15)	[13]
12	C16:0_BF_	18.56-19.59	3	4.2 × 10^-4^	−0.43 (0.12)	[26]
13	C16:0_BF_	9.60-9.62	2	1.5 × 10^-4^	−0.54 (0.14)	New
		24.49-25.37	3	9.2 × 10^-5^	−0.54 (0.14)	New
14	C16:0_BF_	14.96-15.17	2	5.0 × 10^-4^	−0.42 (0.12)	[12]
		90.64-93.33	5	2.1 × 10^-4^	0.50 (0.13)	New
15	C14:0_BF_	122.99-124.15	3	1.7 × 10^-5^	−0.05 (0.01)	New
16	C14:0_BF_	4.95-5.01	3	7.2 × 10^-5^	0.08 (0.02)	[27]
		69.40-70.12	2	1.6 × 10^-5^	0.08 (0.02)	[29]
18	C14:0_BF_	27.04-27.20	3	1.2 × 10^-5^	0.06 (0.01)	[30]

*P*-value and additive effects of the most significant SNP in each interval; *a* (SE): additive effect (standard error); ^§^Reference number of the previous studies identifying a QTL for the same trait in any fat tissue.

**Table 5:** **Chromosomal Regions with Trait Associated SNPs (TAS) affecting IMF fatty acid (FDR = 0.05)**

SSC	Trait	Chromosomal region (Mb)	N° of TAS	***P***-value	***a*** (SE)	Ref^§^
1	C16:0_IMF_	52.52-84.70	106	8.5 × 10^-6^	−0.57 (0.12)	[11]
		102.64-103.05	3	2.8 × 10^-4^	−0.75 (0.20)	[11]
	C18:2_IMF_	83.14-87.07	3	1.4 × 10^-4^	0.96 (0.29)	[11, 12]
		103.89-112.25	4	1.8 × 10^-4^	0.89 (0.29)	[11, 12]
		122.39-125.33	7	1.6 × 10^-4^	0.97 (0.31)	[11, 12]
		137.22-141.52	23	4.2 × 10^-5^	−1.03 (0.28)	[11, 12]
4	C16:0_IMF_	96.10-97.84	6	6.0 × 10^-4^	−0.72 (0.21)	[11, 13, 23]
	C16:1(n-7)_IMF_	54.27-59.91	7	2.6 × 10^-5^	0.21 (0.04)	[11, 23]
		96.38-96.48	2	1.1 × 10^-5^	0.23 (0.05)	[11, 23]
		136.10-136.33	3	1.7 × 10^-7^	0.21 (0.03)	[11]
	C18:2_IMF_	13.60-13.89	2	5.0 × 10^-5^	−0.99 (0.28)	[9, 10, 23, 24]
		19.84-21.78	4	4.1 × 10^-5^	1.23 (0.32)	[9, 10, 23, 24]
		27.68-34.65	14	5.0 × 10^-6^	−1.10 (0.26)	[9, 10, 23, 24]
		31.92-47.70	24	2.4 × 10^-6^	−1.11 (0.25)	[9, 10, 23, 24]
		54.27-54.69	17	2.6 × 10^-5^	−0.92 (0.25)	[9, 10, 23, 24]
		59.06-73.18	30	1.4 × 10^-5^	−1.14 (0.29)	[9, 10, 23, 24]
		73.18-74.58	2	7.5 × 10^-4^	0.80 (0.28)	[9, 10, 23, 24]
5	C14:0_IMF_	28.55-30.13	5	6.3 × 10^-6^	0.08 (0.02)	[11]
8	C16:0_IMF_	90.73-92.44	5	2.1 × 10^-4^	0.55 (0.14)	[9, 11, 12, 25]
		99.33-99.49	3	9.7 × 10^-6^	0.57 (0.12)	[9, 11, 12, 25]
		110.90-111.01	2	1.4 × 10^-5^	0.65 (0.14)	[9, 11, 12, 25]
		114.21-121.04	17	7.7 × 10^-9^	−0.74 (0.12)	[9, 11, 12, 25]
		123.81-125.08	2	1.7 × 10^-4^	0.53 (0.14)	[9, 11, 12, 25]
		130.63-134.57	3	7.7 × 10^-6^	0.60 (0.13)	[9, 11, 12, 25]
	C16:1(n-7)_IMF_	99.33-99.49	3	3.4 × 10^-7^	0.21 (0.04)	[9, 11, 25]
		114.24-122.29	18	4.9 × 10^-9^	−0.23 (0.04)	[9, 11, 25]
11	C18:1(n-9)_IMF_	4.65-4.81	3	2.0 × 10^-5^	1.69 (0.43)	[11]
	C18:2_IMF_	4.65-5.10	4	1.2 × 10^-4^	−1.25 (0.36)	[11]
13	C16:0_IMF_	109.42-113.53	7	4.8 × 10^-5^	−0.54 (0.14)	New
	C18:2_IMF_	110.18-114.48	6	2.1 × 10^-4^	−1.08 (0.34)	New
14	C18:2_IMF_	1.47-13.67	26	9.8 × 10^-6^	1.22 (0.31)	[11–13]
		51.55-53.07	7	3.8 × 10^-4^	−0.96 (0.33)	[11–13]
15	C16:0_IMF_	106.39-109.72	5	5.2 × 10^-4^	−0.80 (0.23)	[13]
		122.99-124.17	5	2.1 × 10^-5^	−0.60 (0.16)	[13]
17	C16:0_IMF_	22.32-26.80	3	1.1 × 10^-4^	−0.59 (0.15)	[11]

*P*-value and additive effects of the most significant SNP in each interval; *a* (SE): additive effect (standard error); ^§^Reference number of the previous studies identifying a QTL for the same trait in any fat tissue.

Pleiotropic TAS regions were identified following the decision tree of the statistical contrasts shown in Figure 2b. Subsequently to the pleiotropic TAS region identification (*q*-value < 0.05) by GWAS using a model fitting pleiotropic effects on a particular FA, in both fat tissues, the most significant SNPs of each region were tested against their respective single effects in BF or IMF. The results of LR tests revealed six significant pleiotropic TAS regions shown in Table 6 with their corresponding position indicated in Mb. These regions correspond to the positions in genetic distance: 47.85-48.43 cM on SSC1, 67.92-73.92 cM on SSC4 and 58.08-93.60 cM on SSC8 for C16:0_BF_ and C16:0_IMF_, 66.24-66.96 cM on SSC4 for C18:2_BF_ and C18:2_IMF_ and 74.88-75.36 cM and 81.36-93.60 cM on SSC8 for C16:1(n-7)_BF_ and C16:1(n-7)_IMF_.

**Table 6:** **Significant chromosomal Regions with TAS using a** ***pleiotropic*** **GWAS model (FDR = 0.05)**

SSC	Trait	Chromosomal region (Mb)	N° of TAS	***P***-value	***a*** (SE)
1	C16:0_BF_	81.21-84.71	65	1.1 × 10^-5^	−0.52 (0.15)
	C16:0_IMF_				−0.62 (0.15)
4	C16:0_BF_	94.00-99.01	16	3.2 × 10^-4^	−0.50 (0.14)
	C16:0_IMF_				−0.45 (0.15)
	C18:2_BF_	92.05-92.44	5	1.8 × 10^-5^	0.83 (0.19)
	C18:2I_MF_				0.90 (0.29)
8	C16:0_BF_	83.84-130.62	115	1.0 × 10^-11^	−1.00 (0.10)
	C16:0_IMF_				−0.71 (0.11)
	C16:1(n-7)_BF_	99.33-99.49	3	1.1 × 10^-9^	0.13 (0.03)
	C16:1(n-7)_IMF_				0.21 (0.04)
	C16:1(n-7)_BF_	110.90-126.88	34	1.1 × 10^-11^	−0.16 (0.02)
	C16:1(n-7)_IMF_				−0.23 (0.04)

*a* (SE): additive effect (standard error).

### *Targeted eQTL mapping and association analyses of Longissimus dorsi gene expression data*

According to the previous linkage results, an eQTL scan focused on chromosomal regions showing tissue-consistent effects on FA was conducted with *Longissimus dorsi* gene expression data, one of the tissues with fatty acid composition data. The analyzed regions corresponded to the intervals of 80–110 cM in SSC8, 1–20 cM in SSC11 and 53–63 cM in SSC17. Additionally, in order to reduce the number of conducted tests, the analyses were performed for 470 preselected probes representing genes related to fatty acid metabolism. Putative eQTLs were considered those whose confidence interval brackets the target gene position on the chromosome [31]. Although 13 eQTLs were identified at nominal *P-*value <0.005 (Additional file 1: Table S1), only one eQTL for the *MGST2* gene expression at 84 cM on SSC8 (nominal *P-*value = 0.3 × 10^-4^; *a* = −0.46 ± 0.10) reached the established false discovery ratio (FDR = 20%), calculated from the effective number of expression probes (Neff = 207.45) and the number of tested positions for each one of the analyzed chromosome regions (30, 20 and 10, respectively).

Similarly to the eQTL, association analyses for *Longissimus dorsi* gene expression data were conducted focusing on the four TAS regions showing tissue-consistent results according to the previous GWAS, pleiotropic pTAS regions. These regions corresponded to the intervals 81.21-84.71 Mb of SSC1 (containing 65 SNPs), 92.05-92.44 Mb and 94.00-99.01 Mb of SSC4 (containing 5 and 16 SNPs, respectively) and 83.84-130.62 Mb (containing 115 SNPs) of SSC8. Putative gene expression TAS (eTAS) were considered those where the TAS region bracket the target gene position on the chromosome. A total of 94 eTAS regions were identified at nominal *P-*value < 0.005 (Additional file 2: Table S2), eight of them reached the assumed false discovery ratio (FDR = 20%), calculated from the effective number of expression probes and the effective number of SNPs for each one of the analyzed TAS regions (Meff = 12.5, 1.58, 23.98 and 89.01, respectively). Two eTAS regions in SSC1 showed effects on *LIPG* and *LDLR* genes expression, two in SSC4 on *RDH16* and *NUDT7* and four in SSC8 on *MGST2, KIT, IL1R2* and *ELOVL6* genes (Table 7).

**Table 7:** **Significant chromosomal Regions with TAS affecting gene expression in** ***Longissimus dorsi***

					Annotation		
Chromosome	Region (Mb)	N° of SNPs	***a*** (SE)	***P***-value	Probe	Gene	Chromosome (Position, Mb)
1	81.21-85.72	9	0.72 (0.18)	6.7 × 10^-5^	Ssc.21663.1.A1_at	*LIPG*	1 (108.64)
1	84.36-85.72	2	−0.48 (0.11)	9.8 × 10^-6^	Ssc.21926.1.S1_at	*LDLR*	2 (70.19)
4	92.44-92.69	2	0.48 (0.13)	5.7 × 10^-4^	Ssc.22641.3.S1_at	*RDH16*	5 (24.13)
4	92.04-92.44	5	−0.57 (0.14)	1.2 × 10^-4^	Ssc.11186.1.S1_at	*NUDT7*	6 (10.10)
8	114.73-123.96	26	−0.67 (0.14)	1.3 × 10^-6^	Ssc.1008.1.A1_at	*ELOVL6*	8 (120.12)
8	117.90-126.37	28	0.38 (0.07)	1.6 × 10^-6^	Ssc.21635.1.A1_at	*MGST2*	8 (92.87)
8	117.90-126.37	29	−0.43 (0.08)	1.1 × 10^-7^	Ssc.16096.2.S1_a_at	*KIT*	8 (43.55)
8	120.94-128.08	15	−0.64 (0.12)	8.8 × 10^-7^	Ssc.25357.1.S1_at	*IL1R2*	15 (54.94)

*a* (SE): additive effect (standard error).

**Figure 3:**
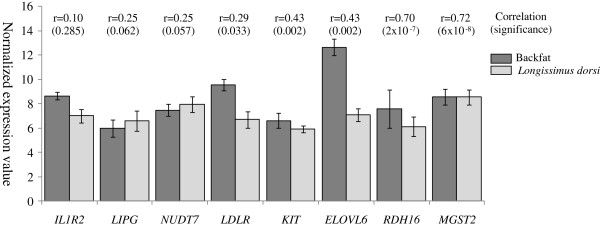
**Expression values in** ***Longissimus dorsi*** **and backfat of the genes for which eQTL or eTAS regions were identified.** Graphical representation of the mean and standard deviation of the microarray normalized gene expression values measured in *Longissimus dorsi* and backfat samples of 40 backcrossed pigs. Pearson correlations and significance were calculated on these 80 expression data.

Since the gene expression analyses were focused on those regions showing pleiotropic effects on the FA content of both analyzed tissues, and as the limited number of available BF gene expression data (40 samples) did not allow us to conduct linkage or association analyses, correlations between IMF and BF gene expression measures were calculated. The correlation values for the genes affected by eQTL or eTAS in the previous analyses are shown in Figure 3. Significant and positive correlations between their expression levels were observed for *LDLR, KIT, ELOVL6, RDH16* and *MGST2* genes. These results may indicate a common expression regulatory element for these genes in both tissues.

## Discussion

In the present study a whole-genome scan based on high-density genotyping has been conducted following different strategies, QTL scan by linkage and GWAS, in order to identify chromosomal regions with significant tissue-specific and tissue-consistent effects on the FA composition of two fat deposits, intramuscular fat and backfat. The justification of this double approach arises from the different hypothesis about the causative mutations underlying each method. The linkage QTL scan is based on the assumption of alternative alleles fixed in each parental line of the experimental intercross. However, the GWAS is free of this assumption about frequencies, and the detected associations are based on the linkage disequilibrium between SNPs and causative mutations. When the frequencies of causative mutations are not largely divergent in the p arental lines, the linkage QTL analyses lose detection power, and therefore GWAS would contribute to the identification of chromosomal regions undetected in the linkage QTL scan. In the current study, several chromosomal regions affecting FA composition were detected by both approaches, as the regions identified on SSC4, SSC8 and SSC14 affecting diverse FA in BF or the identified ones on SSC4, SSC8, SSC11 and SSC14 affecting also several FA in IMF. However, other significant genome regions were identified only by one of the two approaches. Particularly remarkable were the associations found on SSC1 affecting several FA in both BF and IMF which were undetected by the linkage scan, and the QTL region on SSC17 at 58 cM affecting C16:0 content in BF and IMF, undetected by GWAS. Moreover, the great majority of the regions with pleiotropic effects on a particular FA in both fat deposits were detected only by one of the approaches, except those identified on SSC8 for C16:0 and C16:1(n-7) acids. These results show that both approaches can provide complementary results, as reported in previous studies [32].

A comparison of the results obtained for both fat depots showed that in general those obtained in BF were more numerous and significant than those detected in IMF. In fact the QTL and TAS regions detected on SSC8, which affected C16:0_BF_, were the most significant of the scans carried out in both fat deposits. Several previous studies revealed that the heritability estimates of fatty acid composition are lower in IMF than in other fat depots, pointing out a larger environmental component for IMF fatty acid composition than for other tissues [7, 8]. Even so, the analyses conducted in the present study have allowed us to confirm the existence of several tissue-consistent regions showing effects on fatty acid composition. Most of the detected QTLs and TAS regions were tissue-specific, supporting the hypothesis that the major part of the genetic basis of fatty acid composition differs among fat tissues.

Joining linkage QTL and GWAS results, over 30 chromosomal regions showed association with fatty acid composition specifically in BF. Although many regions with effects on fatty acid composition in BF have already been reported in previous studies (porcine QTL database, http://www.animalgenome.org/cgi-bin/QTLdb/SS/index), new associations in 11 of the 18 autosomes are reported here for the first time (Table 8). Likewise, over 20 chromosomal regions showed association with fatty acid composition specifically in IMF. Several regions detected by linkage and association analyses in this fat depot had already been identified in a previous GWAS study conducted on the same animal material by Ramayo-Caldas et al. [11] or in other previous studies using different animal material [9, 10, 12–14, 23–27, 29, 30] but six new regions in four different chromosomes are reported here for the first time (Table 8). Slight differences in the position of the overlapping QTL regions can be observed between Ramayo-Caldas et al., [11] study and the present one likely due to the different porcine assembly version and marker numbers employed in each study. The *Sscrofa10* assembly version and 48,119 SNPs were employed in the study of Ramayo-Caldas et al. [11], while re-annotation of the SNPs with the updated *Sscrofa10.2* assembly version and a selected set of 14,503 SNPs have been employed in the present study.

**Table 8:** **Summary of the new QTLs for fatty acid composition identified in the current study**

SSC	Position Mb	Trait	Tissue	Methodology
Single QTL regions				
1	48.24-49.05	C20:3	BF	GWAS
3	9.44-13.53	C18:0	IMF	Linkage
3	15.71-17.67	C18:2	IMF	Linkage
6	8.01-8.06	C14:0	BF	GWAS
7	6.01-8.65	C17:0	BF	Linkage
8	99.33-99.49	C14:0	BF	Linkage/GWAS
8	110.90-125.08	C14:0	BF	Linkage/GWAS
8	91.57-92.43	C20:3	BF	Linkage/GWAS
8	99.33-99.49	C20:3	BF	Linkage/GWAS
8	117.67-120.10	C20:3	BF	Linkage/GWAS
8	122.07-124.10	C20:3	BF	Linkage/GWAS
11	23.15-23.20	C16:0	BF	GWAS
11	11.98-15.27	C17:0	BF	Linkage
11	11.98-12.68	C20:3	IMF	Linkage
12	37.00-42.66	C16:0	BF	Linkage
12	2.51-4.04	C17:0	BF	Linkage
13	9.60-9.62	C16:0	BF	GWAS
13	24.49-25.37	C16:0	IMF	GWAS
13	110.18-114.48	C18:2	IMF	GWAS
14	90.64-93.33	C16:0	BF	GWAS
15	122.99-124.15	C14:0	BF	GWAS
16	9.56-16.53	C16:1	BF	Linkage
17	40.42-45.39	C16:0	BF/IMF	Linkage
17	41.27-45.39	C20:3	BF	Linkage
Pleiotropic QTL regions				
1	81.21-84.71	C16:0	BF/IMF	GWAS
4	94.00-99.01	C16:0	BF/IMF	GWAS
	92.05-92.44	C18:2	BF/IMF	GWAS
8	83.84-130.62	C16:0	BF/IMF	Linkage/GWAS
	99.33-99.49	C16:1	BF/IMF	Linkage/GWAS
11	11.98-12.68	C20:3	BF/IMF	Linkage
17	41.27-41.82	C16:0	BF/IMF	Linkage

The current study has mainly focused on identifying chromosomal regions showing tissue-consistent effects for fatty acid composition measured in both BF and IMF. The results of the present study allowed us to identify for the first time with a formal pleiotropic statistical approach seven different pleiotropic regions with effects on particular fatty acids in both fat deposits. Moreover, the conducted complementary eQTL scans have allowed us to highlight particular candidate genes.

The tissue-consistent region identified on SSC1 for C16:0 fatty acid content was detected by GWAS analyses. The frequencies of the most significant SNP within the TAS region were far from the hypothesis of alternative alleles in the parental generation: the *H3GA0002028G* allele was fixed in the founder Iberian boars and at a frequency equal to 0.75 in the founder Landrace sows. This TAS region, that expands 3.5 Mb and contains 65 SNPs with significant effects, revealed also an association with the gene expression in *Longissimus* of two important genes (*LDLR, LIPG*) with respective functional roles in lipid transport and HDL catabolism. Moreover, a positive significant correlation was detected for muscle and backfat *LDLR* expression, suggesting that a common expression regulatory element of this gene could be acting in both tissues, in accordance to the hypothesis of pleiotropic effects. Because the *LDLR* gene is mapped at 70.19 Mb on SSC2, a potential regulator of *LDLR* transcription should be mapping within the SSC1 pleiotropic region under study. Regulatory binding sites in the *LDLR* gene promoter have been described for C/EBPbeta, AP-2alpha isoform 3, AP-1, AP-2alpha isoform 2, PPAR-gamma1, PPAR-gamma2, AP-2alpha isoform 4, AP-2alpha, AP-2alphaA and SP1 transcription factors (http://www.genecards.org). However, none of them has been mapped within or even close to the target region up to now.

The two tissue-consistent regions identified on SSC4 for C16:0 and C18:2 fatty acids were also detected by GWAS analyses. For these two pTAS regions, the most significant SNP alleles, *ASGA0087140C* and *MARC0090207G,* were fixed in the founder Iberian boars and displayed 0.48 and 0.63 frequencies in the founder Landrace sows, respectively, as well far from the hypothesis of alternative frequencies in the parental populations. The region showing effects on C16:0 (94.00-99.01 Mb), revealed no association with gene expression in *Longissimus dorsi*. By contrast, the SSC4 region showing effects on C18:2 (92.05-92.44 Mb), provided association with the expression of *RDH16* and *NUDT7* genes. Besides, a high positive correlation (0.70) was found between muscle and backfat *NUDT7* expression. The *NUDT7* gene encodes for a peroxisomal coenzyme A diphosphatase whose function, among others, is to remove potentially toxic oxidized CoA disulfide from peroxisomes to maintain the capacity for beta-oxidation of fatty acids. Although *NUDT7* gene maps on other chromosome different than the pleiotropic region under studio, the promoter region of this gene contains a known binding site for transcription factor Pbx1 (http://www.genecards.org). The *PBX1* gene maps very close to the target region, at 93.57 Mb in SSC4. Therefore, a mutation on *PBX1* could potentially modify *NUDT7* gene expression, altering the peroxisome capacity for fatty acids oxidation. Further structural studies focused on *PBX1* gene would allow us to determine the mutation providing *NUDT7* expression changes, potentially responsible for the C18:2 contents in both fat deposits.

The most reliable results were found in SSC8, where linkage QTL scan as well as GWAS revealed significant pleiotropic regions with effects on C16:0 and C16:1(n-7) fatty acids. Moreover, the multitrait QTL linkage analysis allowed us to statistically determine for the first time the presence of a region with pleiotropic effects in both fat deposits with a maximum at 90 cM affecting C14:0_BF_ , C16:0_BF_ , C16:1(n-7)_BF_ , C16:0_IMF_ and C16:1(n-7)_IMF_. In both fat tissues, the Iberian *Q* allele would increase the content of SFA and MUFA and decrease the PUFA content. The effects found are consistent with those described by Clop et al. [9] and Sánchez et al. [12] on C16:0 but only in BF. In the current study, the effects have been detected in both tissues and not just on C16:0 but also on C16:1(n-7). In addition, the same pleiotropic regions revealed significant associations with the expression *in Longissimus dorsi* of three interesting genes *ELOVL6, MGST2* and *KIT*, in addition to significant positive correlations between their expression values in loin muscle and backfat. The only significant eQTL identified corresponded to this chromosomal region on SSC8. Among these genes, *MGST2* and *ELOVL6* fall within or very close to the target pleiotropic region (92 and 120 Mb, respectively). The *MGST2* gene encodes a microsomal glutathione S-transferase, a protein that is involved in the biosynthesis of leukotrienes and prostaglandin E from arachydonic fatty acid (C20:4) [33]. The role of the *ELOVL6* in fatty acid metabolism is even clearer, as it codes for a fatty acid elongase which catalyzes the elongation of FAs with 12–16 carbons to C18 [34]. Moreover, in a previous study conducted on the same animal material [17], the authors detected a strong effect of *ELOVL6*:c.-533C > T SNP on C16:0 and C16:1 (n-7) percentages measured in BF and IMF. Furthermore, significant differences in *ELOVL6* gene expression were observed when animals were classified by the *ELOVL6*:c.-533C > T genotype in BF tissue, but not in *Longissimus* muscle. Despite the strong association of *ELOVL6*:c.-533C > T polymorphism with the BF gene expression and with C16:0 and C16:1(n-7) percentages, the authors identified a stronger association of another SNP, *ALGA0049135*, outside but near to the *ELOVL6* gene. All these results, together with the obtained in the present study, seem to indicate that the mutation underlying the QTL would be located in a regulatory element close to the *ELOVL6* gene, affecting *ELOVL6* gene transcription and other nearby genes, such as *MGST2* and even the *KIT* gene. Regulatory binding sites in the *ELOVL6* promoter have been described for SREBP-1, MLX, HNF4-gamma, KLF10, ESRR-alpha and SP1 transcription factors [17]. However, none of them has been mapped within or even close to the SSC8 target region up to now. Therefore, further studies focused on possible regulatory elements around the *ELOVL6* gene region would allow us to determine the actual causal mutation underlying this QTL.

Finally, two other tissue-consistent regions were identified on SSC11 for C20:3 and on SSC17 for C16:0 by linkage QTL scans. Even if significant eQTL at nominal *P*-value were detected on these pQTL regions, no eQTLs remained significant after multiple test correction.

Although the candidate genes proposal has been based on gene expression data, it should be taken into account that the causal mutation does not necessarily provide gene expression changes. Therefore for those regions were significant eQTL or eTAS regions could not be detected, strong positional and functional candidate genes can be highlighted. For instance, the *RGS4* gene maps within the confidence interval of SSC4 region (at 95.09 Mb) and it constitutes a strong functional candidate gene for that pTAS region (94.00-99.01 Mb). The *RGS4* gene codes for a member of the large family of RGS proteins that participate in several physiological processes. Specifically, RGS4 controls the balance between adipose tissue lipolysis and lipogenesis through fatty acid and glucose homeostasis [35]. Similarly, the *TRIB3* gene maps within the confidence interval of SSC17 QTL (at 39.52 Mb) and also constitutes a strong functional candidate gene for this QTL region (39–42 Mb). The *TRIB3* gene codes for a tribbles protein, which has been associated with the control of fatty acid synthesis and insulin resistance as well as regulating plasma triglyceride and HDL cholesterol levels in human species. Several mechanisms of molecular action have been proposed for the tribbles mediated control of these processes, including the regulation of signaling events, protein turnover and transcription [36]. Further structural studies of *RGS4* and *TRIB3* genes would help in the identification of the mutations underlying the quoted QTL effects in SSC4 for C16:0 and SSC17 for C16:0 content, respectively.

In spite of the limited number of animals employed in the present study, we have obtained consistent results, confirmed and identified new QTL regions for FA composition in BF and IMF, pleiotropic effects in both fat tissues and consistent gene expression results that allowed us to better understand the genetic basis of the fatty acid composition in porcine.

## Conclusions

The complementary genome scans conducted in the present study have allowed us to confirm several chromosome regions previously associated to fatty acid composition in backfat and intramuscular fat, but even more, to identify 24 new ones on SSC1, SSC3, SSC6, SSC7, SSC8, SSC11, SSC12, SSC13, SSC14, SSC15, SSC16 and SSC17. Although most of the detected regions were tissue-specific, supporting the hypothesis that the major part of the genes responsible for FA composition differs among tissues, seven chromosomal regions with tissue-consistent effects were detected on SSC1 for C16:0, on SSC4 for C16:0 and C18:2, on SSC8 for C16:0 and C16:1(n-7), on SSC11 for C20:3 and on SSC17 for C16:0. The complementary eQTL scans focused on the identified tissue-consistent regions have allowed us to identify some powerful candidate genes and target regions to be responsible for these pleiotropic effects, as the *PBX1* transcription factor and a transcription regulatory element close to *ELOVL6* gene.

## Methods

### Animals and phenotypic records

Animals from a backcross belonging to the IBMAP experimental population were used [18, 37]. The F1 was obtained from the cross between three Iberian Guadyerbas boars and 30 Landrace sows. Five F1 boars were coupled with 25 Landrace sows to obtain 157 backcrossed animals (BC). The percentage of 15 BF fatty acids and 16 IMF fatty acids were measured by gas chromatography in BF samples taken between the third and the fourth ribs and in 200 g of *Longissimus dorsi* samples, in the 157 BC animals (Additional file 3: Table S3). Animal manipulations were performed according to the Spanish Policy for Animal Protection RD1201/05, which meets the European Union Directive 86/609 about the protection of animals used in experimentation.

### SNP data

**Figure 4:**
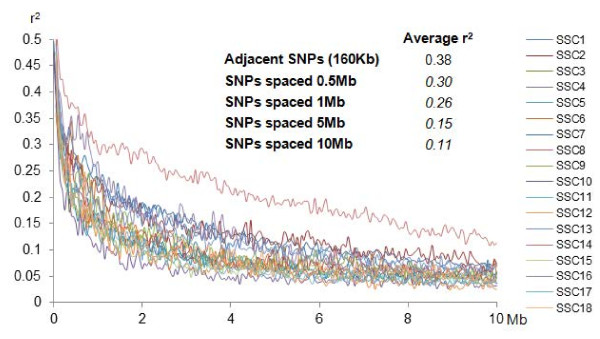
**Linkage disequilibrium across porcine autosomes from backcross animals.** Graphical representation of the average linkage disequilibrium (r^2^) between markers in the backcross animals at various distances ranging from 0 to 10 Mb. Average r^2^ for SNPs with adjacent positions (160 Kb), SNPs spaced 0.5 Mb, 1 Mb, 5 Mb and 10 Mb apart.

The 157 BC animals and their ancestors from F1 and F0 generations of the IBMAP experimental cross were genotyped with the PorcineSNP60 Genotyping Bead Chip (Illumina) using the Infinium HD Assay Ultra protocol (Illumina) [16]. Raw individual data had high-genotyping quality (call rate >0.99). The SNPs were filtered according to our previous study [28]. Briefly, those SNPs displaying call rates less than 0.85, a minor allele frequency less than 0.15, non-Mendelian inheritance and located in sex chromosomes or not mapped in the Sscrofa10assembly were removed. A total number of 14,503 SNPs (mean distance 0.16 Mb or 0.24 cM) were retained in the dataset and employed in the GWAS. Linkage disequilibrium (LD) map (Figure 4) shows high LD in all the autosomes, as expected by the experimental design. The LD value between adjacent SNP pairs (r^2^ = 0.38) is similar to the reported for six commercial European lines by Veroneze et al. [38], who these LD values enough for whole- genome studies. A selection of the most informative SNPs was carried out based in their genetic distance and according to the linkage maps generated in our previous study [28]. One of each group of contiguous SNPs with genetic distance equal to zero was retained as representative of the linkage group for subsequent analyses, a total of 8,417 SNPs were retained and used in the linkage QTL analyses. In a posterior step, the SNP sets used for the different analyses were re-annotated with the updated version *Sscrofa10.2* porcine genome assembly.

### Genome-wide scans

A genome-wide classical QTL scan was performed using the linkage maps built in our material in a previous study [28] and 8,417 SNPs evenly mapped across the 18 autosomes with the mean distances reported in Fernández et al. [39]. The first and basic QTL scan was performed in intervals of 1 cM, using the following single QTL and tissue specific single trait model:

where *y*_*ijk*_ is the *ijk-*th individual record of the percentage of a particular FA on the fat of one of the analyzed tissues (BF or IMF); *S*_i_ and *B*_j_ are the systematic effects for gender (male or female) and slaughter batch (three in total); *β* is a covariate coefficient with *CW*_*ijk*_ being the carcass weight; *a*_*QTL*_ is the QTL additive effect; *P*_*aijk*_ is the additive coefficient calculated as *P*_*aijk*_*= Pr(QQ) − Pr(qq)*, the probability of the *ijk*-th individual being homozygous for alleles of Iberian origin minus the probability of being homozygous of alleles of Landrace origin; *u*_*ijk*_ is the infinitesimal genetic effect; and *e*_*ijk*_ is the random residual. The infinitesimal genetic effect was considered as random, with covariance *Aσ*^*2*^_*u*_, *A* being the numerator relationship matrix.

Moreover, a series of analyses was carried out using bivariate models fitting single or two QTLs in the same chromosome, according to the decision tree represented in Figure 2a, for checking possible pleiotropic single QTL affecting the content of a particular FA in both tissues and other alternative hypothesis. The bivariate models were similar to the basic model nevertheless the (co)variances of the infinitesimal genetic effects are *A* ⊗ , where ⊗ denotes the Kronecker product and the *y* and *z* subindices correspond to the two traits, the respective content of each FA in BF and IMF. The bivariate single QTL model represents a QTL with pleiotropic effects in both tissues, and the bivariate two QTLs model represents the hypothesis of two different QTLs on the same chromosome affecting the content of the same FA in each tissue.

Genome-wide association analyses of the 14,503 SNPs mapped along the 18 autosomes were separately performed for the content of every FA on each tissue. The following standard animal model was used:

where *λ*_*ijkl*_ is an indicative variable related with the number of copies of one of the alleles of the *l*-th SNP, which takes values of 1 or −1 when the *ijk*-th animal was homozygous for each allele or 0 if the animal was heterozygous; *g*_*SNP*_ represents the additive effect of *l*-th SNP. Complementary analyses were carried out using bivariate association models, according to the decision tree represented in Figure 2b, for checking possible pleiotropic SNP affecting the content of a particular FA in both tissues.

Likelihood ratio tests (LR) for QTL or SNP effects were separately calculated comparing for every cM or SNP the appropriate full and reduced models. The nominal *P*-values were calculated assuming a χ2 distribution of LR values with the degrees of freedom given by the difference between the number of estimated parameters in the full and reduced models. All these analyses were performed using the Qxpak v5.1 software [40]. The 95% confidence intervals (CI) of the location of classical QTLs were calculated following Mangin et al. [41]. The procedure of Storey et al. [42], based on the distribution of nominal *P*-values resulting from the multiple LR tests, was used for controlling the false discovery rate (FDR) in every achieved genome scan at a desired level of FDR = 0.05.

Relations between physical and linkage distances were set up based in our previous linkage map study [28].

### Targeted eQTL mapping

The microarray expression data of *Longissimus dorsi* muscle samples from 102 BC individuals were obtained using Gene Chip Porcine Genome Arrays (Affymetrix, Boston, MA, USA). Similar expression data of backfat samples were also obtained from 40 of these BC individuals. Total RNA extraction, microarray hybridization, and scanning were performed according to Affymetrix protocols as described by Cánovas et al. [21]. Expression data were generated with Affymetrix GCOS 1.1.1 software. Microarray data quality evaluation was carried out with AffyPLM software in the Bioconductor Package (http://www.bioconductor.org). Data normalization to reduce technical variations between chips through GeneChipRobust Multi-Array Average algorithm was conducted with BRB-ArrayTools software (http://linus.nci.nih.gov/BRB-ArrayTools.html). Moreover, a “minimum fold-change” filter implemented in the BRB-ArrayTools was applied in order to select only probes displaying more than 20% of expression values over ± 1.5 times the median expression of all the 102 arrays of muscle samples. Expression probe annotation was performed according to the updated version of Tsai et al. [43]. Ingenuity Pathways Analysis (IPA, https://analysis.ingenuity.com/pa) was used in order to search for predefined pathways and functional categories related to fatty acid or lipid metabolism. Additionally, a complementary analyses using DAVID database was carried out to investigate their functional implications and preselecting probes which correspond to genes related to fatty acid metabolism. A total of 485 probes were preselected, but in those cases where there was more than one probe representing the same gene only the probe with the highest expression mean was chosen.

As result of this targeted procedure, the expression data on *Longissimus dorsi* of 470 selected genes were used to map eQTL both by linkage and SNP association analyses. The analyses were conducted using the aforementioned respective basic models described for phenotypic QTL (pQTL), being now *y*_*ijk*_ the expression value of each one of the probes in the *ijk-*th individual. Genetical genomics techniques assume that gene expression levels are affected by the polymorphisms affecting the trait of interest, and we focused this study exclusively on the chromosome regions with detected pleiotropic pQTL. Linkage mapping of eQTL was limited at positions spaced one cM within the CI of each identified pleiotropic pQTL, and the SNP association tests of expression values were only performed for the SNPs included in the chromosome regions of pleiotropic trait associated SNPs (TAS).

Testing for putative QTL positions in 470 expression traits produced a lot of nominal *P*-values that required multiple test corrections. As the performed tests were not independent, the effective number of probes and the effective number of marker tests by TAS were calculated according to Nyholt [44] using the alternative equivalent formula proposed by Moskvina et al. [45]. Finally, the procedure of Benjamini and Yekutieli [46] was used for controlling the FDR at a desired level of 0.20.

The correlation values between the expression levels in *Longissimus* and backfat samples of the genes affected by eQTLs were also calculated.

### Availability of supporting data

The complete microarray data set is available at Gene Expression Omnibus (GEO) under accession number GSE52626 (http://www.ncbi.nlm.nih.gov/geo/query/acc.cgi?acc=GSE52626)

## Electronic supplementary material

Additional file 1: Table S1: Significant eQTL (nominal *P*-value <0.005) for *longissimus dorsi.* (XLSX 10 KB)

Additional file 2: Table S2: Chromosomal Regions with TAS affecting gene expression in *longissimus dorsi* (nominal *P*-value <0.005). (XLSX 19 KB)

Additional file 3: Table S3: Fatty acid composition of BF and IMF measured in (Iberian x Landrace) x Landrace pigs. (XLSX 12 KB)

## Abbreviations

FA: Fatty acid
SFA: Saturated fatty acid
MUFA: Monounsaturated fatty acid
PUFA: Polyunsaturated fatty acid
QTL: Quantitative trait locus
SSC: Sus scrofa chromosome
SNP: Single nucleotide polymorphism
GWAS: Genome-wide association studies
BF: Backfat
IMF: Intramuscular fat
BC: Backcross
eQTL: Expression quantitative trait locus
eTAS: Expression trait-associated SNPs
Neff: Effective number of expression probes
Meff: Effective number of SNPs
LR: Likelihood ratio
TAS: Significant trait-associated SNPs
LPL: *Lipoprotein lipase*
LIPG: *Lipase, endothelial*
RDH16: *Retinol dehydrogenase 16 (all-trans)*
NUDT7: *Nudix (nucleoside diphosphate linked moiety X)-type motif 7*
IL1R2: *Interleukin 1 receptor type II*
MGST2: *Microsomal glutathione S-transferase 2*
ELOVL6: *Fatty acid elongase 6*
PBX1: *Pre-B-cell leukemia homeobox 1*
C/EBPbeta: CCAAT/enhancer binding protein (C/EBP), beta
AP-1: Jun proto-oncogene
AP-2: Transcription factor AP-2 alpha (activating enhancer binding protein 2 alpha)
PPAR: Peroxisome proliferator-activated receptor
SP1: Trans-acting transcription factor 1
SREBP-1: Sterol regulatory element binding transcription factor 1
MLX: MAX dimerization protein
HNF4: Hepatocyte nuclear factor 4
KLF10: Kruppel-like factor 10
ESRR: Estrogen-related receptor
RGS4: *Regulator of G-protein signaling 4*
TRIB3: *Tribbles homolog 3.*
